# Visual Awareness in Binocular Rivalry Modulates Induced Pupil Fluctuations

**DOI:** 10.5334/joc.16

**Published:** 2018-02-08

**Authors:** Immo Schütz, Johanna Elisabeth Busch, Lukas Gorka, Wolfgang Einhäuser

**Affiliations:** 1 Physics of Cognition Group, Institute of Physics, Chemnitz University of Technology, Chemnitz, DE

**Keywords:** Vision, Eye movements, Attention, Visual perception, Consciousness

## Abstract

When a visual stimulus oscillates in luminance, pupil size follows this oscillation. Recently, it has been demonstrated that such induced pupil oscillations can be used to tag which stimulus is covertly attended. Here we ask whether this “pupil frequency tagging” approach can be extended to visual awareness, specifically to inferring perceptual dominance in Binocular Rivalry between complex stimuli. We presented two distinct stimuli, a face and a house, to each eye and modulated their luminance at 1.7 Hz either in counter-phase (180° phase shift), with a 90° phase shift or in phase (0° control). In some conditions, we additionally asked observers to attend either of the stimuli. The luminance modulation was sufficiently subtle that rivalry dynamics did not differ among these conditions, and was also indistinguishable from unmodulated presentation of the stimuli. For the 180° and the 90° phase-shifted stimuli, we found that the phase of the pupil response relative to the stimuli was modulated by perceptual dominance; that is, the relative phase depended on the stimulus the observer was aware of. In turn, this perceptually dominant stimulus could be decoded from the phase of the pupil response significantly above chance. Neither percept dependence of the phase nor significant decoding was found for the 0° control condition. Our results show that visual awareness modulates pupil responses and provide proof of principle that dominance in rivalry for complex stimuli can be inferred from induced pupil fluctuations.

## 1. Introduction

The pupil light response is not only affected by the actual illuminance incident on the eye, but also by various cognitive influences. For example, the pupil reacts to the expected rather than the actual luminance level of a given scene (Laeng & Endestad, 2012; Naber & Nakayama, 2013), to predicted light levels of imagined scenes (Laeng & Sulutvedt, 2014), to words related to brightness and darkness (Mathôt, Grainger, & Strjikers, 2017) as well as to the luminance of covertly attended stimuli (Binda & Murray, 2015; Binda, Pereverzeva, & Murray, 2013; Mathôt, van der Linden, Grainger & Vitu, 2013). This latter effect has been exploited to measure covert attentional selection through pupil size to an extent that spatial mapping of attention through pupillometry is now a realistic possibility (Tkacz-Domb & Yeshurun, 2017). Inspired by the technique of steady-state visual evoked potentials (ssVEP), which has been used to measure attention with EEG (Morgan, Hansen, & Hillyard, 1996), “pupil frequency tagging” (PFT) has recently been suggested as a robust method to track attention using pupillometry, albeit at lower frequencies: Naber, Alvarez and Nakayama (2013) simultaneously modulated the brightness of multiple images sinusoidally at different frequencies between 0.7 and 3.2 Hz. In the induced oscillations in pupil size, power at the specific frequency of a stimulus increased when it was attended. This allowed for decoding the attended scene from the power spectrum of the pupil-size time course. Using a related approach, Mathôt, Melmi, van der Linden and van der Stigchel (2016) instructed participants to covertly attend one of two stimuli flickering in counterphase. The attended stimulus could be determined based on the phase lag between pupil size and the modulation of each stimulus. Mathôt and colleagues demonstrated that this approach allows users to convey responses to binary questions (e.g., „yes“ and „no“ or selection between sets of letters) at a speed and accuracy useful for communication in this pupil-based human-computer interface. In the present study, we ask whether such induced pupil fluctuations are not only modulated by attention but also by visual awareness in Binocular Rivalry (BR) between complex stimuli.

In BR, distinct stimuli are presented to the left and right eye. Access to visual awareness then typically alternates between the two stimuli despite the physically constant stimulation (Dutour, 1760, translated by O’Shea, 1999; Wheatstone, 1838), in a way that is characteristic of many multistable perceptual phenomena (see Blake, 2001 for a review). Pupil size has been found to increase around the times of perceptual alternations for many of these multistable phenomena, including auditory multistability and BR (Einhäuser, Stout, Koch, & Carter, 2008; Hupé, Lamirel, & Lorenceau, 2009; Kietzmann, Geuter, & König, 2011; Kloosterman, Meindertsma, van Loon, Lamme, Bonneh & Donner, 2015; Naber, Frässle, & Einhäuser, 2011). Moreover, the pupil light response has been used to track awareness to brighter or darker stimuli in monocular rivalry and BR of grating stimuli (Naber et al., 2011; Fahle, Stemmler & Spang, 2011). Here we extend this pupil-based tracking of visual awareness to BR of complex visual stimuli, specifically the rivalry between a face and a house image that is widely used in fMRI studies of BR (cf. Tong, Nakayama, Vaughan & Kanwisher, 1998).

For the question whether pupil size is not only modulated by attention but also awareness to be of relevance, it is critical to note that attention and awareness can be dissociated (see Koch & Tsuchiya, 2007, for a review). This dissociation notwithstanding, attention has profound influences on BR. For example, attentional deployment influences switching rates in BR (Paffen, Alais & Verstraten, 2006), and focusing attention on a specific stimulus can increase perceptual dominance of the corresponding stimulus (Marx & Einhäuser, 2015; Ooi & He, 1999; van Ee, van Dam & Brouwer, 2005). Beyond the theoretical relevance of a possible modulation of pupil dynamics by awareness, the question is also of practical relevance for the further development of no-report paradigms to study visual awareness (see Tsuchiya, Wilke, Frässle, & Lamme, 2015 for a review), especially whether pupil-based no-report paradigms can in principle be extended from simple gratings (Fahle et al., 2011; Naber et al., 2011) to more complex stimuli without interfering with the rivalry dynamics as such.

Here we adapt the PFT method to test whether visual awareness can also modulate pupil-size fluctuations induced by oscillating stimuli. Specifically, we modulated a house and a face image (cf. Tong et al., 1998), presented dichoptically, sinusoidally in luminance. The phase difference of the luminance modulations between the two stimuli was either 180° or 90° (experimental conditions) or 0° (control condition). In some versions of the experimental conditions, we also asked the observers to explicitly attend one of the stimuli. If the current percept influences the pupil response, we expect that the phase of the pupil time course relative to the oscillations of the stimuli depends on the currently dominant percept for the experimental conditions, and does so irrespective of the observers’ attentional instruction.

## 2. Methods

### 2.1. Participants

Twelve participants (5 female, 7 male) between the ages of 20 and 39 (mean age: 27) volunteered for the experiment. All participants had normal or corrected-to-normal vision and intact stereoscopic vision. Participants gave written informed consent and received either course credits or monetary compensation for their participation. All procedures were performed in accordance with the Declaration of Helsinki and were determined by the applicable ethics committee (Ethikkommission, Fakultät für Human- und Sozialwissenschaften, TU Chemnitz) to not require in-depth ethics evaluation (Az. V122-WET-NoReport-22012016).

### 2.2. Experimental Setup and Stimuli

Stimuli were presented dichoptically on two 21-inch CRT displays (Samsung SyncMaster 1000DF; Samsung, Seoul, Korea) at a resolution of 1280 × 1024 pixels and a frame rate of 85 Hz. CRTs were arranged for stereoscopic presentation (Wheatstone, 1838), with each screen visible to one eye only at a viewing distance of 30 cm through a set of infrared-transparent („cold“) mirrors angled at 45° (Naber et al., 2011). Eye movements and pupil size were recorded through the mirrors using an infrared camera-based eye tracking device (EyeLink 1000; SR-Research, Ottawa, ON, Canada) at a sampling rate of 1000 Hz. Data from the left eye were analyzed. Participants’ responses were collected using a game controller (Microsoft SideWinder USB; Microsoft, Redmond, WA, USA) and recorded together with eye-tracking data. Stimulus presentation and eye tracking were controlled using MATLAB (TheMathWorks, Natick, MA, USA) and the Psychophysics-(Brainard, 1997; Kleiner et al., 2007) and Eyelink toolboxes (Cornelissen, Peters & Palmer 2002).

**Figure 1:**
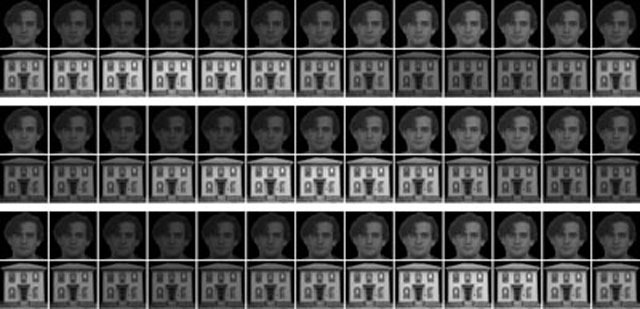
Stimuli and luminance modulations used. Each row represents one presentation cycle (360° or 2π, corresponding to 0.588s at 1.7 Hz in steps of 30° (π/6)). In this example, the face image always represents the left and the house image the right eye. Note that the luminance modulation depicted here is only approximate depending on screen/printer calibration; in the actual experiment modulation was adjusted to be sinusoidal in luminance by correcting for display Gamma (γ = 2.26). Top: phase shift of 180° (π; counterphase). Middle: phase shift of 90° (π/2). Bottom: same phase (control).

During the experiment, distinct images (a house and a face, similar to stimuli used in Tong et al. (1998), see also Figure 1) were presented dichoptically to the participants. The face image was selected from a database for face-recognition research (Phillips, Wechsler, Huang & Rauss, 1998) and the house image was acquired from an online photo sharing website (flickr.com) under a license permitting noncommercial use. Images were presented centrally in each eye at a resolution of 200 × 200 pixels (subtending 11.8 × 11.8°), with the association of images to eyes counterbalanced across subjects and conditions. A random-dot mask positioned around the images (size 600 × 600 pixels, 32.0 × 32.0°) and a central fixation cross (size 10 × 10 pixels, 0.6 × 0.6°) were presented to both eyes to aid in focusing on a constant depth plane.

### 2.3. Experimental Paradigm

The experiment consisted of seven blocks of 5 minutes each. Before each block, the eye tracker was calibrated using a 9-point calibration grid. In all blocks, participants were instructed to press and hold one button while they perceived the house as dominant and another when perceiving the face as dominant. If a combination of both images was perceived, they were instructed to hold down both buttons until a single percept again emerged as dominant. For the pupil analysis, we only consider periods of exclusive dominance of either house or face; that is, periods in which exactly one button was held.

**Figure 2:**
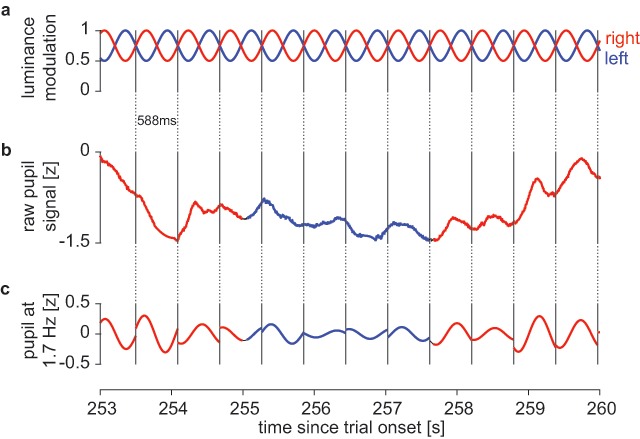
Analysis of individual presentation cycles. Example data from one observer (luminance modulated condition at 180° phase difference; observer #3, block 2). Top: luminance modulation presented to the left (blue) and right eye (red). Middle: Raw z-standardized pupil trace. Colors indicate the percept reported by button press (blue: image in left eye, red: right eye). Bottom: F1 Fourier component of pupil signal at 1.7 Hz. Colors as in middle panel.

In total, five different *luminance-modulation* conditions and two *static conditions* were presented during the experiment. In the *static conditions*, both images were displayed statically and at constant luminance for the whole block duration (300s). In all other conditions, stimulus luminance was modulated in each eye between half and full image luminance by multiplying each image with a shifted and scaled 1.7 Hz sine wave (each presentation cycle comprising 50 display frames of 11.8ms at 85 Hz screen refresh rate) ranging from 0.5 to 1.0 (Figure 2A). To ensure sinusoidal modulation in luminance (rather than in pixel-value/luma), the modulation was taken to the power of the monitor’s inverse gamma (1/γ with γ = 2.26). One condition modulated both images with zero phase difference (*same-phase luminance-modulated 0° control*). In the other *luminance modulated* conditions, images were modulated at the same frequency but at a phase difference of either 180° (π) or 90° (π/2), with the stimulus in the left eye leading. These experimental luminance-modulation conditions were split further in *naïve* and *attention-instruction* conditions. The same luminance manipulation was applied in both, but in *attention-instruction* participants additionally received auditory instructions to attend one of the images without moving their gaze, starting with either *house* or *face* and alternating every 30s. Instructions („house“, „face“) were generated using text-to-speech software and presented via headphones. For all observers, the first and the last block were a static condition (hereafter: *initial static* and *final static*), which served to verify that alternation dynamics were constant throughout the experiment. The fourth block was the *luminance-modulated control* (*0°*) *condition* for all observers. Since the *naïve* conditions should precede the *attention-modulation* conditions, the former were assigned to blocks 2 and 3, the latter to blocks 5 and 6, and the order of 180° and 90° luminance modulation was counterbalanced across observers (Table 1). In one half of participants, the initial static block presented the house to the left and face to the right eye, and vice versa in the other half. The assignment of images to eyes then alternated with each new block to avoid potential image-level effects.

**Table 1:** Order of presentation conditions.

Block	Condition name	Description (details see text)

1	initial static	house and face presented statically
2–3 (counterbalanced)	180° modulation (naïve)	face and house luminance-modulated in counterphase
	90° modulation (naïve)	face and house luminance-modulated with 90° phase shift, left eye leading
4	0° luminance-modulation control	both stimuli luminance-modulated in phase
5–6 (counterbalanced)	180° modulation (attention instruction)	face and house luminance-modulated in counterphase, instruction to attend either face or house alternating in 30s intervals
	90° modulation (attention instruction)	face and house luminance-modulated with 90° phase shift, left eye leading, instruction to attend either face or house, alternating in 30s intervals
7	final static	house and face presented statically

### 2.4. Data Processing

Eye-tracking data and button-press data were imported into MATLAB for further processing. Blinks were detected using the manufacturer’s built-in algorithm for saccade and blink detection at default parameters. Visual inspection showed that some blink artifacts remained, which were obvious as extremely rapid apparent changes in pupil size. We removed all data points for which the change in pupil dilation to the next data point exceeded 5 standard deviations of this change rate. Additionally, 50 ms of data were removed before and after each detected blink, and the missing data due to blinks was interpolated using cubic spline interpolation. Timeseries for each block were z-standardized (mean of zero, standard deviation of one). Statistical analysis was performed in MATLAB and R. An alpha level of .05 was used for all analyses, effect size measures are reported as suggested by Lakens (2013) for a within-subjects design. Data and code to replicate figures and analyses are available for download at https://doi.org/10.5281/zenodo.1006000.

### 2.5. Analysis

#### 2.5.1. Behavioral Responses

Median dominance durations for each stimulus type (face/house) were determined for each presentation condition. Potential influences of the presentation condition and stimulus type on dominance durations were assessed by a 2 (stimulus type) × 7 (condition) repeated measures ANOVA. Since we expected interaction effects between stimulus type and condition a priori only for the attention-instruction condition (and thus no overall interaction for the 2 × 7 ANOVA, where the attention-instruction condition only represents a minor fraction of conditions), the attention-instruction conditions were also analyzed separately using paired t-tests for the factor stimulus type.

#### 2.5.2. Dominant percept per presentation cycle

For analysis of induced pupil fluctuations, we chunked the z-normalized pupil data into the 588ms (1/1.7 Hz) presentation cycles, such that each period for the left eye contained one period of a sinusoid starting at phase –90° (Figures 1 and 2A, blue trace). Depending on the condition, the luminance modulation for the right eye was shifted by 180°, 90°, or 0° (control). If the pupil responds more strongly to the dominant percept, a consistent phase shift should become apparent for periods in which the stimulus of the right eye is reported dominant as compared to the left eye (Figure 2B). Note that the pupil response should be inverted relative to the luminance modulation (lower luminance implying larger pupil size) and also shifted (to account for the delay in the response). Since the overt button response also lags relative to the change in percept at a similar order of magnitude, we did not correct for either. We performed a fast Fourier transform for each separate presentation cycle and consider the value (hereafter referred to as “Z”) at the presentation frequency of 1.7 Hz (the “F1 component”, Figure 2C) further. The imaginary part of Z corresponds to the sinusoidal, the real part to the cosinusoidal (i.e., phase shifted by 90°) component of the pupil size in the given presentation cycle. If the phase of the pupil depends on the percept, Z will consistently differ between the reported percepts. For simplicity, we restricted statistical analysis to the imaginary part of Z, since we expected the difference to occur mostly on the sinusoidal phase. Note that even in a highly idealized linear noise-free situation, 0°, 90° and 180° conditions would yield different oscillation amplitudes if either percept contributed with a hypothetical fixed weight. (For example, if one percept contributes to the pupil oscillation with 90% and the other with 10%, this would result in a 91% amplitude in the 90° condition, and an 80% amplitude in the 180° condition; for equal weights, 90° would yield an amplitude of 71% (\sqrt {0.5}), while 180° would yield 0 [obviously with no difference between percepts in either case]). Hence, the amplitude of pupil oscillations, and thus the amplitude of Z, are not directly comparable between different modulation conditions. Therefore, we separately tested for each condition, whether – for example–the imaginary part of Z differed between left-eye reported and right-eye reported by means of a two-sided paired t-test.

#### 2.5.3. Percept prediction in each individual per presentation cycle

In addition to testing whether there is a significant difference in Z between periods of left and right eye dominance, we analyzed how well Z predicted the percept in an individual presentation cycle by adapting a measure from signal detection theory. To this end, we defined a threshold for the imaginary part of Z and counted how frequently right eye dominance was correctly predicted as right eye dominance (hits), misclassified as left eye dominance (misses), how frequently left eye dominance was classified as left eye dominance (correct rejections) or left eye dominance was misclassified as right eye dominance (false alarms). By varying the threshold, we obtain a receiver operating characteristics (ROC) curve for each individual and condition. The area under this curve (AUC) is a measure of discrimination performance. To calculate an upper estimate for possible decoding performance, in addition to applying this analysis to the imaginary part of Z, we also determined the optimal AUC across all phase angles (i.e., weighing between imaginary and real part) in the complex plane for each individual and condition.

## 3. Results

### 3.1. Behavioral Responses

Over all seven conditions, observers reported exclusive dominance of the house image for 31.8% (SD: 9.4%) and of the face image for 36.1% (SD: 9.4%) of the total experimental time. The remainder accounted for mixed percepts (28.7%, SD: 16.9%) and no reported percept (3.5%, SD: 2.9%). The median duration of exclusive dominance periods amounted to 2.89s (SD: 1.29s) for face dominance and 2.65s (SD: 1.24s) for house dominance. We found no main effect of the stimulus type (face/house) on dominance durations (*F*(1, 11) = 1.58, *p* = .24, η*²_G_* = .01). Neither did we find a main effect of condition (*F*(6, 66) = 1.37, *p* = .24, η*²_G_* = .02), nor an interaction between these factors (*F*(6, 66) = 0.35, *p* = .91, η*²_G_* = .004). When participants were instructed to try and attend the house stimulus, they reported (not necessarily exclusive) perception of the house in 68.6% (SD: 9.2%) of the time, while they reported face only in 56.9% (12.2%) of the time (*t*(11) = 2.88, *p* = .01, *d_s_* = 1.18). Similarly, if instructed to attend the face stimulus, they reported dominance of the face 74.4% (11.8%) and of the house 51.4% (9.5%) of the time (*t*(11) = 5.91, *p* < .001, *d_s_* = 2.41).

### 3.2. Phase of the pupil signal indicates perception

**Figure 3:**
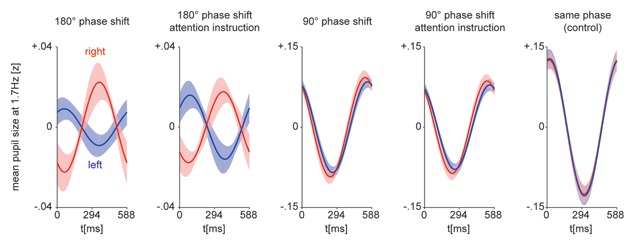
Pupillary response at 1.7 Hz (F1 component) over the duration of one presentation cycle, averaged over participants for all luminance modulation conditions and same phase control. Colors indicate reported percept (blue: left eye image, red: right eye image), shaded bands indicate +/–1 SEM.

**Figure 4:**
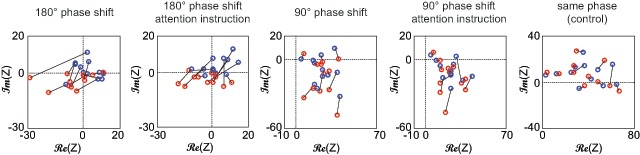
Complex plane representation of F1 component, averaged across presentation cycles for each participant and luminance modulation condition. Blue circles indicate reported percept of left eye image, red circles report of right eye image. Lines connect opposing perceptual reports within each individual.

Participants reported on average 182.7 presentation cycles in which the left-eye stimulus was perceived exclusively (SD: 63.5, range 86–270) and 171.2 presentation cycles in which the right-eye stimulus was perceived exclusively (SD: 43.5, range 95–227). Overall, 4246 cycles of 588ms duration with exclusive perceptual reports (70% of all presented cycles) were analyzed for phase differences. When separating these presentation cycles and averaging the F1 component at presentation frequency (Figure 3), we find a nearly 180° phase shift reflected in the pupil for the 180° luminance-modulation conditions (Figure 3, left), a slight phase difference for the 90° luminance-modulation conditions (Figure 3, middle) and virtually no difference in the 0° control condition (Figure 3, right). We quantify this difference by averaging the complex F1 component (Z) for each individual and luminance-modulation condition and plotting these in the complex plane (Figure 4). For all experimental luminance-modulation conditions (90°, 180°, naïve and attention-instruction), but not for the luminance-modulation control (0°) condition, we find a clear and consistent separation between presentation cycles in which the left eye stimulus (blue) or the right eye stimulus (red) was perceived. We again quantify this difference by comparing the imaginary part of Z between reported percepts (left/right eye) for each condition across individuals, and find a significant difference in all experimental luminance-modulation conditions, but not the control (0°) condition (180° without attention instruction: *t*(11) = 3.84, *p* = .003, *d_s_* = 1.57; with attention instruction *t*(11) = 4.10, *p* = .002, *d_s_* = 1.67; 90°: *t*(11) = 2.87, *p* = .02, *d_s_* = 1.17 and *t*(11) = 2.35, *p* = .04, *d_s_* = 0.96; control: *t*(11) = 0.53, *p* = .61, *d_s_* = 0.21; Figure 4).

### 3.3. Moment-by-moment prediction of perceptual state

To test how well the induced pupil fluctuations can predict an observer’s perception on a moment-by-moment basis, we computed the AUC for the discrimination in each individual and condition, using the imaginary part of Z for each presentation cycle. We found AUCs significantly above chance (50%) for all luminance-modulation conditions except the control (180° without attention instruction: *t*(11) = 4.54, *p* < .001, *d_s_* = 1.86; with attention instruction *t*(11) = 4.50, *p* < .001, *d_s_* = 1.84; 90°: *t*(11) = 3.07, *p* = .01, *d_s_* = 1.25 and *t*(11) = 2.46, *p* = .03, *d_s_* = 1.00; control: *t*(11) = 0.69, *p* = .50, *d_s_* = 0.28). Despite all experimental conditions reaching significance, numerically, the AUCs are not far above chance when only comparing along the imaginary axis (means by condition: 180°/naive: 54.7%, 180°/instruction: 55.0%, 90°/naive: 56.0%, 90°/instruction: 54.5%, same phase control: 51.1%). If the optimal axis for discrimination in the complex plane is selected for each individual and condition by calculating all possible rotations in steps of 1° and selecting the maximal AUC, the across-subject averages reach up to 62.5% and individual values up to 78.7%.

## 4. Discussion

We show that changes in visual awareness in binocular rivalry affect induced pupil-size fluctuations in accordance with the subjective report of participants. Importantly, this was achieved by luminance modulations that were sufficiently subtle to induce no significant change to rivalry dynamics as such, and irrespective of whether the observers reported their percept naively or were instructed to try and make one percept dominant. Consequently, we show for the first time an effect of awareness on the modulation of the pupil light response for complex visual stimuli.

One objective of applying luminance modulation to high-level stimuli is the development of a no-report paradigm for such complex stimuli in BR. Although moment-by-moment decoding in the present paradigm is far below optimal and also clearly below other oculomotor measures such as the optokinetic nystagmus (OKN; Einhäuser, Thomassen & Bendixen, 2017; Fox, Todd, & Bettinger, 1975; Frässle et al., 2014; Wilbertz, Ketkar, Guggenmos, & Sterzer, 2017; Marx & Einhäuser, 2015; Naber et al., 2011; Soltész, Pastukhov, Braun, & Kovács, 2016), decoding performance is significantly above chance for all experimental conditions. While further optimization is necessary, it is conceivable that the notion of no-report paradigms in BR can be extended to complex stimuli and used in combination with other techniques, in particular fMRI. Along this line, it has already been demonstrated that combining fMRI with OKN yields better decoding than either method alone (Wilbertz, Ketkar, Guggenmos, & Sterzer, 2017).

The pupil-frequency-tagging method we used here to track visual awareness in BR is reminiscent of ssVEP in EEG (Morgan, Hansen, & Hillyard, 1996). ssVEPs have indeed been successfully used to measure visual awareness in BR: flickering each image with a specific frequency, Brown & Norcia (1997) could decode the dominant stimulus from the EEG power spectrum, and a similar approach is also possible with MEG (Tononi, Srinivasan, Russell, & Edelman, 1998). Unlike ssVEP approaches and Naber et al. (2013) in pupillometry, we here chose phase differences akin to Mathôt et al. (2016), since pilot studies indicated that some observers were substantially affected in their rivalry dynamics when different frequencies were chosen for stimuli presented to different eyes. While a visual “flicker effect” is also clearly noticeable in our stimuli, we find no effect of the luminance modulation on reported awareness dynamics. Although the presentation of spatially identical stimuli that flicker at different frequencies does not cause rivalrous phenomena by itself (O’Shea & Blake, 1986), temporal modulations of visual stimuli have previously been associated with changes in perceptual switching dynamics (Breese, 1899), thus our null-finding here is non-trivial. Of course, it is conceivable – and even likely – that more subtle effects of the luminance manipulation on behavior do exist and were not detected in the present study. Post-hoc sensitivity analysis indicates that, at our sample size and an assumed power of .8, the corresponding effect size would have to be at least *d* = 0.88 for the influence to be reliably detected (*d* = 1.14 for a power of .95). For the present proof-of-principle study that primarily concerns the pupillometric effects and does not focus on the behavioral data, we do not consider this an issue. Depending on the specific research question that is to be addressed using the pupillometric method introduced here, the limits on acceptable behavioral effects introduced by the pupil oscillation and therefore the required power might differ.

The main difference between ssVEPs and pupil frequency tagging is the range of available frequencies. The range of possible modulation frequencies in pupillometry is limited by the slow pupillary response (Alexandridis & Manner, 1977). While our particular choice of frequency (1.7 Hz) was in part motivated by having the cycle length a simple integer multiplier (50) of the screen’s inverse refresh rate (85 Hz), it is well within the range for optimal decoding of attentional selection in an earlier study (Naber et al., 2013), which shows substantial performance decline starting at about 2 Hz. In contrast, ssVEP frequencies can be chosen above the (foveal) flicker fusion frequency and thus remain unnoticeable to the observer. Despite these obvious advantages of EEG and MEG, pupillometry is comparably simple in its application, which might render it advantageous for application in specific populations and for the combination with other techniques.

Exploiting the pupil light response to tag awareness in rivalry has to be distinguished from the pupil dilation induced by perceptual alternations as such (Einhäuser et al., 2008). While the latter is probably related to the sympathetic system acting to consolidate perception (cf. Aston-Jones & Cohen, 2005; Bouret & Sara, 2005), the former presumably modulates the pupil light response, which is mediated by the parasympathetic system (cf. Loewenfeld, 1993). Unlike earlier studies which used pupillometry to tag the currently dominant stimulus (Naber et al., 2011; Fahle et al., 2011), the current approach does not require the two stimuli to be intrinsically different in luminance. As such, it lends itself to a broader class of rivaling stimuli, including the face/house rivalry employed here.

To conclude, our results indicate that frequency tagging of high-level stimuli can be used to induce phase differences in the pupillary response that represent subjective percept in Binocular Rivalry. In its present form, this method can already be used to verify the veridicality of button-press reports within an experimental session. Moreover, moment-by-moment decoding results indicate that, with optimization of parameters or the combination with other techniques, this paradigm might also allow for decoding of subjective percepts in BR of high-level stimuli.

## Data Accessibility Statement

Data and code to replicate figures and analyses are available at https://doi.org/10.5281/zenodo.1006000.

## References

[1] Alexandridis, E., & Manner, M. (1977). Folgefrequenz der Pupille bei flimmernden Lichtreizen. Albrecht von Graefes Archiv für klinische und experimentelle Ophthalmologie, 202(3), 175–180. DOI: 10.1007/BF00407866 301718

[2] Aston-Jones, G., & Cohen, J. (2005). An integrative theory of locus coeruleus-norepineph-rine function: Adaptive gain and optimal performance. Annual Review of Neuroscience, 28, 403–450. DOI: 10.1146/annurev.neuro.28.061604.135709 16022602

[3] Binda, P., & Murray, S. O. (2015). Spatial attention increases the pupillary response to light changes. Journal of Vision, 15(2), 1 DOI: 10.1167/15.2.1 25645434

[4] Binda, P., Pereverzeva, M., & Murray, S. O. (2013). Attention to bright surfaces enhances the pupillary light reflex. Journal of Neuroscience, 33, 2199–2204. DOI: 10.1523/JNEUROSCI.3440-12.2013 23365255 PMC6619119

[5] Blake, R. (2001). A primer on binocular rivalry, including current controversies. Brain and mind, 2, 5–38. DOI: 10.1023/A:1017925416289

[6] Bouret, S., & Sara, S. (2005). Network reset: A simplified overarching theory of locus coeruleus noradrenaline function. Trends in Neuroscience, 28, 574–582. DOI: 10.1016/j.tins.2005.09.002 16165227

[7] Brainard, D. H. (1997). The psychophysics toolbox. Spatial Vision, 10, 433–436. DOI: 10.1163/156856897X00357 9176952

[8] Breese, B. B. (1899). On inhibition. The Psychological Review: Monograph Supplements, 3(1), i–65. DOI: 10.1037/h0092990

[9] Brown, R. J., & Norcia, A. M. (1997). A method for investigating binocular rivalry in real-time with the steady-state VEP. Vision Research, 37, 2401–2408. DOI: 10.1016/S0042-6989(97)00045-X 9381675

[10] Cornelissen, F. W., Peters, E. M., & Palmer, J. (2002). The Eyelink Toolbox: Eye tracking with MATLAB and the Psychophysics Toolbox. Behavior Research Methods, Instruments, & Computers, 34(4), 613–617. DOI: 10.3758/BF03195489 12564564

[11] Dutour, E.-F. (1760). Discussion d’une question d’optique [Discussion on a question of optics]. l’Académie des Sciences. Mémoires de Mathématique et de physique présentés par Divers Savants, 3, 514–530.

[12] Einhäuser, W., Stout, J., Koch, C., & Carter, O. (2008). Pupil dilation reflects perceptual selection and predicts subsequent stability in perceptual rivalry. Proceedings of the National Academy of Sciences of the United States of America, 105, 1704–1709. DOI: 10.1073/pnas.0707727105 18250340 PMC2234208

[13] Einhäuser, W., Thomassen, S., & Bendixen, A. (2017). Using binocular rivalry to tag foreground sounds: Towards an objective visual measure for auditory multistability. Journal of Vision, 17(1), 34 DOI: 10.1167/17.1.34 28129418

[14] Fahle, M. W., Stemmler, T., & Spang, K. M. (2011). How Much of the “Unconscious” is Just Pre – Threshold? Frontiers in Human Neuroscience, 5, 120 DOI: 10.3389/fnhum.2011.00120 22025912 PMC3198031

[15] Fox, R., Todd, S., & Bettinger, L. A. (1975). Optokinetic nystagmus as an objective indicator of binocular rivalry. Vision Research, 15, 849–853. DOI: 10.1016/0042-6989(75)90265-5 1154667

[16] Frässle, S., Sommer, J., Jansen, A., Naber, M., & Einhäuser, W. (2014). Binocular rivalry: frontal activity relates to introspection and action but not to perception. Journal of Neuroscience, 34, 1738–1747. DOI: 10.1523/JNEUROSCI.4403-13.2014 24478356 PMC6827584

[17] Hupé, J. M., Lamirel, C., & Lorenceau, J. (2009). Pupil dynamics during bistable motion perception. Journal of Vision, 9(7), 10 DOI: 10.1167/9.7.10 19761325

[18] Kietzmann, T. C., Geuter, S., & König, P. (2011). Overt visual attention as a causal factor of perceptual awareness. PLoS One, 6(7), e22614 DOI: 10.1371/journal.pone.0022614 21799920 PMC3143177

[19] Kleiner, M., Brainard, D., Pelli, D., Ingling, A., Murray, R., & Broussard, C. (2007). What’s new in psychtoolbox-3. Perception, 36(14), 1–16.

[20] Kloosterman, N. A., Meindertsma, T., van Loon, A. M., Lamme, V. A. F., Bonneh, Y. S., & Donner, T. H. (2015). Pupil size tracks perceptual content and surprise. European Journal of Neuroscience, 41, 1068–1078. DOI: 10.1111/ejn.12859 25754528

[21] Koch, C., & Tsuchiya, N. (2007). Attention and consciousness: Two distinct brain processes. Trends in Cognitive Sciences, 11, 16–22. DOI: 10.1016/j.tics.2006.10.012 17129748

[22] Laeng, B., & Endestad, T. (2012). Bright illusions reduce the eye’s pupil. Proceedings of the National Academy of Sciences of the United States of America, 109(6), 2162–2167. DOI: 10.1073/pnas.1118298109 22308422 PMC3277565

[23] Laeng, B., & Sulutvedt, U. (2014). The eye pupil adjusts to imaginary light. Psychological Science, 25(1), 188–197. DOI: 10.1177/0956797613503556 24285432

[24] Lakens, D. (2013). Calculating and reporting effect sizes to facilitate cumulative science: a practical primer for t-tests and ANOVAs. Frontiers in Psychology, 4, 863 DOI: 10.3389/fpsyg.2013.00863 24324449 PMC3840331

[25] Loewenfeld, I. (1993). The pupil: Anatomy, physiology, and clinical applications. Wayne State University Press, Detroit, MI.

[26] Marx, S., & Einhäuser, W. (2015). Reward modulates perception in binocular rivalry. Journal of Vision, 15(1), 15 DOI: 10.1167/15.1.11 25589295

[27] Mathôt, S., Grainger, J., & Strijkers, K. (2017). Pupillary Responses to Words That Convey a Sense of Brightness or Darkness. Psychological Science, 28(8), 1116–1124. DOI: 10.1177/0956797617702699 28613135 PMC5549816

[28] Mathôt, S., Melmi, J. B., van der Linden, L., & van der Stigchel, S. (2016). The Mind-Writing Pupil: A Human-Computer Interface Based on Decoding of Covert Attention through Pupillometry. PLoS One 11(2), e0148805 DOI: 10.1371/journal.pone.0148805 26848745 PMC4743834

[29] Mathôt, S., van der Linden, L., Grainger, J., & Vitu, F. (2013). The Pupillary Light Response Reveals the Focus of Covert Visual Attention. PLoS ONE, 8(10), e78168 DOI: 10.1371/journal.pone.0078168 24205144 PMC3812139

[30] Morgan, S., Hansen, J., & Hillyard, S. (1996). Selective attention to stimulus location modulates the steady-state visual evoked potential. Proceedings of the National Academy of Sciences of the United States of America, 93, 4770–4774. DOI: 10.1073/pnas.93.10.4770 8643478 PMC39354

[31] Naber, M., Alvarez, G. A., & Nakayama, K. (2013). Tracking the allocation of attention using human pupillary oscillations. Frontiers in Psychology, 4, 919 DOI: 10.3389/fpsyg.2013.00919 24368904 PMC3857913

[32] Naber, M., Frässle, S., & Einhäuser, W. (2011). Perceptual rivalry: reflexes reveal the gradual nature of visual awareness. PLoS ONE, 6(6), e20910 DOI: 10.1371/journal.pone.0020910 21677786 PMC3109001

[33] Naber, M., & Nakayama, K. (2013). Pupil responses to high-level image content. Journal of Vision, 13(6), 7 DOI: 10.1167/13.6.7 23685390

[34] Ooi, T. L., & He, Z. J. (1999). Binocular rivalry and visual awareness: The role of attention. Perception, 28, 551–574. DOI: 10.1068/p2923 10664754

[35] O’Shea, R. P. (1999). Translation of Dutour (1760). Retrieved from: https://sites.google.com/site/oshearobertp/publications/translations/dutour-1760.

[36] O’Shea, R. P., & Blake, R. (1986). Dichoptic temporal frequency differences do not lead to binocular rivalry. Perception & Psychophysics, 39, 59–63. DOI: 10.3758/BF03207584 3703662

[37] Paffen, C. L. E., Alais, D., & Verstraten, F. A. J. (2006). Attention speeds binocular rivalry. Psychological Science, 17, 752–756. DOI: 10.1111/j.1467-9280.2006.01777.x 16984290

[38] Phillips, P. J., Wechsler, H., Huang, J., & Rauss, P. J. (1998). The FERET database and evaluation procedure for face-recognition algorithms. Image and vision computing, 16, 295–306. DOI: 10.1016/S0262-8856(97)00070-X

[39] Soltesz, P., Pastukhov, A., Braun, J., & Kovacs, I. (2016). Under what conditions is optokinetic nystagmus a reliable measure of perceptual dominance in binocular rivalry? Perception, 45(2), 157.

[40] Tkacz-Domb, S., & Yeshurun, Y. (2017). The size of the attentional window when measured by the pupillary response to light. Journal of Vision, 17(10), 1325–1325. DOI: 10.1167/17.10.1325 PMC6082875 30089801

[41] Tong, F., Nakayama, K., Vaughan, J. T., & Kanwisher, N. (1998). Binocular rivalry and visual awareness in human extrastriate cortex. Neuron, 21, 753–759. DOI: 10.1016/S0896-6273(00)80592-9 9808462

[42] Tononi, G., Srinivasan, R., Russell, D. P., & Edelman, G. M. (1998). Investigating neural correlates of conscious perception by frequency-tagged neuromagnetic responses. Proceedings of the National Academy of Sciences of the United States of America, 95, 3198–3203. DOI: 10.1073/pnas.95.6.3198 9501240 PMC19719

[43] Tsuchiya, N., Wilke, M., Frässle, S., & Lamme, V. A. (2015). No-report paradigms: extracting the true neural correlates of consciousness. Trends in cognitive sciences, 19(12), 757–770. DOI: 10.1016/j.tics.2015.10.002 26585549

[44] van Ee, R., van Dam, L. C. J., & Brouwer, G. J. (2005). Voluntary control and the dynamics of perceptual bi-stability. Vision Research, 45, 41–55. DOI: 10.1016/j.visres.2004.07.030 15571737

[45] Wheatstone, C. (1838). Contributions to the physiology of vision. Part the first. On some remarkable, and hitherto unobserved, phenomena of binocular vision. Philosophical transactions of the Royal Society of London, 128, 371–394. DOI: 10.1098/rstl.1838.0019

[46] Wilbertz, G., Ketkar, M., Guggenmos, M., & Sterzer, P. (2017). Combined fMRI-and eye movement-based decoding of bistable plaid motion perception. NeuroImage, 171, 190–198. DOI: 10.1016/j.neuroimage.2017.12.094 29294388

